# Supplementary material for: MITRE: inferring features from microbiota time-series data linked to host status
Source: Genome Biol. 2019 Sep 2;20:186. doi: 10.1186/s13059-019-1788-y (PMC6721208; doi:10.1186/s13059-019-1788-y)
Supplement: Supplementary file 2 — Table S2. Technical details of the application of MITRE and comparator methods to each classification problem. (PDF 111 kb) [file 13059_2019_1788_MOESM2_ESM.pdf]

Data from study	Variable predicted	max $\hat{R}$	iterations	comparator features	pool
David *et al.* (2014)	Plant-based vs. animal-based diet	1.014	50000	66184; 1344	374848
David *et al.* (2014)	Diet (data renormalized)	1.011	50000	66184; 2096	519954
Kostic *et al.* (2015)	Seroconversion	1.0060	100000	1520; 224	12278
Di Giulio *et al.* (2015)	Premature delivery	1.033	50000	1551; 23	3516
Bokulich *et al.* (2016)	Formula-dominant diet	1.028	50000	25344; 528	397884
Bokulich *et al.* (2016)	Cesarean delivery	1.031	50000	25344; 528	397884
Vatanen *et al.* (2016)	Russian nationality	1.043	25000	18180; 1248	570934
Vatanen *et al.* (2016)	Any allergy	1.036	25000	17646; 1212	555896
Vatanen *et al.* (2016)	Any dietary allergy	1.024	25000	17646; 1212	555896
Vatanen *et al.* (2016)	Egg allergy	1.061	25000	17646; 1212	555896
Vatanen *et al.* (2016)	Dairy allergy	1.068	25000	17646; 1212	555896
Vatanen *et al.* (2016)	Elevated IgE levels	1.032	25000	17646; 1212	555896

Table S2. Technical details of the application of MITRE and comparator methods to each classification problem listed in supplementary table 1, plus the dietary classification problem applied to the data of David *et al.* normalized relative to the abundance of the genus Bacteroides, as shown in Supplementary Figure 1 and discussed in the text. R-hat values are calculated using five independent MCMC chains of the indicated length and represent the minimum value across the parameters monitored (i.e., parameters in the model, except that some high-dimensional vector-valued parameters were downsampled.) 'Pool' is the MITRE detector pool size. The comparator methods were applied both before and after the reduction of the number of variables by the abundance filtering options described in the text and Supplementary Note (reporting the results from whichever choice led to better performance for each method); the applications at these steps led to the larger and smaller numbers of features shown.
